# Vitamin D in Chronic Kidney Disease and Dialysis Patients

**DOI:** 10.3390/nu9040328

**Published:** 2017-03-25

**Authors:** Guillaume Jean, Jean Claude Souberbielle, Charles Chazot

**Affiliations:** 1 NephroCare Tassin Charcot, Sainte Foy les Lyon, 69110, France; chchazot@gmail.com; 2 Service d’explorations fonctionnelles, Hôpital Necker-Enfants malades, AP-HP, Paris 75015, France; jean-claude.souberbielle@aphp.fr; 3 F-CRIN, Investigation Network Initiative-Cardiovascular and Renal Clinical Trialist, Vandoeuvre-lès-Nancy 54500, France

**Keywords:** vitamin D, chronic kidney disease, dialysis, hyperparathyroidism, vitamin D receptor activators

## Abstract

Vitamin D deficiency (<20 ng/mL) and insufficiency (20–29 ng/mL) are common among patients with chronic kidney disease (CKD) or undergoing dialysis. In addition to nutritional and sunlight exposure deficits, factors that affect vitamin D deficiency include race, sex, age, obesity and impaired vitamin D synthesis and metabolism. Serum 1,25(OH)_2_D levels also decrease progressively because of 25(OH)D deficiency, together with impaired availability of 25(OH)D by renal proximal tubular cells, high fibroblast growth factor (FGF)-23 and decreased functional renal tissue. As in the general population, this condition is associated with increased morbidity and poor outcomes. Together with the progressive decline of serum calcitriol, vitamin D deficiency leads to secondary hyperparathyroidism (SHPT) and its complications, tertiary hyperparathyroidism and hypercalcemia, which require surgical parathyroidectomy or calcimimetics. Kidney Disease Outcomes Quality Initiative (KDOQI) and Kidney Disease Improving Global Outcomes (KDIGO) experts have recognized that vitamin D insufficiency and deficiency should be avoided in CKD and dialysis patients by using supplementation to prevent SHPT. Many vitamin D supplementation regimens using either ergocalciferol or cholecalciferol daily, weekly or monthly have been reported. The benefit of native vitamin D supplementation remains debatable because observational studies suggest that vitamin D receptor activator (VDRA) use is associated with better outcomes and it is more efficient for decreasing the serum parathormone (PTH) levels. Vitamin D has pleiotropic effects on the immune, cardiovascular and neurological systems and on antineoplastic activity. Extra-renal organs possess the enzymatic capacity to convert 25(OH)D to 1,25(OH)_2_D. Despite many unanswered questions, much data support vitamin D use in renal patients. This article emphasizes the role of native vitamin D replacement during all-phases of CKD together with VDRA when SHPT persists.

## 1. Background

Vitamin D is a fat-soluble secosteroid that has a specific cytosolic receptor. This hormonal system is involved in the regulation of nearly 3% of the human genome. It was first known to play a central role in calcium and phosphate metabolism; however, more recently, vitamin D deficiency has been associated with numerous events and conditions in the general population such as falls, fractures, diabetes, autoimmune diseases, cardiovascular and renal diseases, tuberculosis, depression, neurodegenerative diseases, and cancer [1,2].

Humans acquire the majority of their vitamin D from sunlight-induced cutaneous synthesis, (approximatively 80%), the rest comes from diet and supplement [1]. Vegetable sources provide ergocalciferol (D_2_) and animal sources provide cholecalciferol (D_3_); both of these have similar metabolisms. These precursors are transported in the liver by a vitamin D binding protein (DBP); then, vitamin D is hydroxylated at the C25 position by specific hydroxylase coded by the CYP2R1 gene. 25-hydroxyvitamin D (25(OH)D) is the main circulating form of vitamin D. It has been considered a precursor of the active form 1,25(OH)_2_D, but, at very high concentration, it has the capacity to bind to vitamin D receptor (VDR). Furthermore, various extrarenal cells express megalin, particularly parathyroid cells and osteoblast, and the 1α-hydroxylase [3]. Therefore, local production of 1,25(OH_2_)D occurred in numerous tissues [4].

Some experts have defined vitamin D deficiency as a serum 25(OH)D level <20 ng/mL and insufficiency as between 21 and 29 ng/mL: a target of >30 ng/mL is suggested for optimal health [1,5,6]. However, this remains controversial because of the lack of a consensus regarding the optimal range for serum 25(OH)D [7,8]. Nevertheless, there is a common understanding that low serum 25(OH)D levels cause a negative calcium balance, secondary hyperparathyroidism (SHPT), and bone disease.

Few foods contain sufficient vitamin D. Therefore, without daily sunlight exposure or fortified food, an important risk for vitamin D deficiency exists. Thus, climate, location, aging, lifestyle, and skin pigmentation affect vitamin D production.

The Workshop Consensus for Vitamin D Nutritional Guidelines estimated that more than 50% of the older population are vitamin D-deficient or vitamin D-insufficient [9]. The same is true for younger populations, even in sunny countries [10]. Therefore, more than one billion people are thought to have vitamin D insufficiency or deficiency worldwide.

In chronic kidney disease (CKD), the hyperphosphaturic osteocyte-derived hormone FGF-23 increases to compensate for phosphate retention and further inhibits renal 1α-hydroxylase expression, and induces the expression of 24-hydroxylase responsible for the degradation of 1,25-(OH_2_)D. However, 24,25(OH)D levels are lower in dialysis patients than in the normal population. Thus, the impaired uptake of 25(OH)D by altered kidneys remains the main cause of 1,25(OH_2_)D deficiency [11] since the metabolic clearance rate of calcitriol seems not altered [12]. In addition to the direct effect of high 25(OH)D levels, local osteoblastic conversion of 25(OH)D to 1,25(OH_2_)D appears to be an important positive regulator of FGF-23 production, particularly in uremia [13].

Together with decreased kidney function, a decrease in 1,25(OH)_2_D leads to hypocalcemia and SHPT, which are the main causes of secondary osteoporosis.

The risk for falls and fracture is four times greater for female dialysis patients compared with the general population [14], and this could justify prevention strategies. Evidence for the usefulness of vitamin D to treat renal bone disease is now nearly six decades old. However, the routine use of vitamin D for preventing or reversing the impact of SHPT on the bones of patients with CKD has been implemented in regular clinical practice for only three decades. During the initial years of dialysis use, native vitamin D compounds (calciferol and calcidiol) were prescribed [15]. In the 1980s, active vitamin D compounds were identified and were used instead [16]. This transition could be explained by the fact that it was assumed that 25(OH)D could not be metabolized to 1,25(OH)_2_D by the kidneys. However, the pharmacological impacts of different vitamin D species and of their different modes of administration cannot be assumed to be uniform across the spectrum.

Both the Kidney Disease Outcomes Quality Initiative (KDOQI) [5] and Kidney Disease Improving Global Outcomes (KDIGO) [17] experts recommend checking and supplementing low serum 25(OH)D levels in CKD and dialysis patients.

The aim of this review was to provide the current actual knowledge and to address the questions nephrologists may have regarding vitamin D in CKD and dialysis patients.

## 2. Why Are Low Serum 25(OH)D Levels Generally Observed in CKD and Dialysis Patients?

CKD represents one of the main conditions associated with low 25(OH)D serum levels. Studies of transplant recipients showed a high frequency of vitamin D insufficiency (>80% of cases) [18]. The same is true for CKD non-transplant patients, with >80% having low serum 25(OH)D levels [19]. Observational studies reported progressive vitamin D deficiency worsening from stage 3 to stage 5D [20]. However, in a cross-sectional study, Guesseous et al. reported that vitamin D deficiency is similarly observed in CKD patients and in the general population [21]. The main causes and risk factors for vitamin D deficiency and insufficiency are displayed in Table 1. In addition, age and female sex, proteinuria, low physical activity [22], diabetes [23], and body adiposity [24] are associated with vitamin D deficiency in CKD patients. Cankaya et al. reported that serum vitamin D level is lower in peritoneal dialysis (PD) and hemodialysis (HD) patients compared with CKD and renal transplant patients [25].

Jacob et al. reported that chronic hemodialysis patients exhibit defective photoproduction of cholecalciferol, despite normal epidermal content of substrate, 7-dehydrocholesterol [28].

In transplant recipients, the same factors are found together with the risk identified when prescribing calcineurin inhibitors treatments [18].

VDR levels are reduced and CYP24A1 is increased in CKD patients [26]. In addition, vitamin D binding protein (VDBP) seems to increase and is not involved in 25(OH)D deficiency in CKD patients [30]. Takemoto et al. reported that 25(OH)D tubular reabsorption is impaired due to decreased renal megalin [27]. As a compensatory mechanism, vitamin D catabolism, measured by 24,25(OH)_2_D, is decreased in CKD patients and especially in dialysis patients [31]. Michaud et al. suggested that uremia decreases 25(OH)D synthesis secondary to PTH-mediated reduction in liver CYP450 isoforms [29]. Therefore, it could be speculated a vicious cycle involving vitamin D and SHPT that requires progressive amounts of vitamin D compounds to be reversed.

## 3. Consequences of Low Serum 25(OH)D Levels in CKD and Dialysis Patients 

The associations between vitamin D deficiency and insufficiency and symptoms or outcomes are reported in Table 2.

Low 25(OH)D has been associated with high bone turnover, SHPT, and decreased bone mineral density (BMD) in CKD and dialysis patients [32,33,34]. Low 25(OH)D has been associated with muscle weakness [35] and risk of falls [36]. Boudville et al. reported that 25(OH)D deficiency is associated with muscular weakness and falls in dialysis patients, but with a J curve and maximal benefit in the range between 24 and 44 ng/mL of serum 25(OH)D levels [36]. Vitamin D deficiency has been associated with metabolic syndrome and obesity [37] in HD patients. In PD patients, low vitamin D levels have been associated with cognitive impairment [42]. In transplant recipients, low serum 25(OH)D level is associated with a rapid decline in renal function [46,47]. Vitamin D deficiency has been associated with insulin resistance, ventricular hypertrophy, atherosclerotic disease [38], and vascular calcifications [39]. London et al. reported an inverse relationship between arterial stiffness and serum 25(OH)D and 1,25(OH)_2_D levels in dialysis patients [41]. Ravani et al. reported that serum 25(OH)D levels <15 ng/mL are associated with both the risk for mortality and progression to dialysis in predialysis CKD patients [43]. A resistance to vitamin D_3_ has been reported in CKD and is associated with progression of renal disease [48]. Besides, the defects in calcitriol upregulation of renal Klotho expression may play a role in the progression of renal damage and cardiovascular disease in CKD patients [49,50].

The renal protective effect of vitamin D has been linked with inhibition of the renin-angiotensin system and NF-κB pathway [51] and upregulation of nitric oxide synthase transcription in vascular endothelial cells [52].

In a meta-analysis [44], Pilz et al. reported that 10 ng/mL higher 25(OH)D level was associated with a decrease of 14% in mortality risk. The prognosis of CKD patients seems to improve with vitamin D supplementation [53]. Low 1,25(OH)_2_D [54] and low 25(OH)D [45] have been associated with mortality in dialysis patients. In some dialysis cohorts, a clear association between low serum 25(OH)D levels and mortality has been reported. For example, in a French cohort, mortality risk was increased by 30% when the serum 25(OH)D level was <18 ng/mL [45]. However, the relationship between outcomes and serum 25(OH)D level should be interpreted together with serum PTH and FGF-23 [55].

## 4. How Can Vitamin D Deficiency and Insufficiency Be Supplemented?

Because of the long life of complex 25(OH)D and DBP (480 h), daily (1000 U D_3_), weekly [56] or monthly (40,000 U D_3_) [57] regimens seem efficient for restoring 25(OH)D levels [58]. For years, we chose to provide monthly cholecalciferol during dialysis to insure observance [59]. Zitt et al. reported that a weekly dosing regimen of 100 U/kg body weight for dialysis patients allows achievement of target serum 25(OH)D level in only 27% of cases [60]. Vitamin D provided during dialysis is more effective than home prescriptions [61]. Calcifediol is sometimes prescribed as daily, biweekly, or monthly vitamin D supplementation with the same efficiency [62]. In France, nephrologists used to prescribe cholecalciferol as oral 100,000 IU monthly doses, which allow normalization of serum 25(OH)D level in >85% of cases [59]. In Belgium, Delanaye et al. reported their experience using oral cholecalciferol 25,000 IU every two weeks, which allowed achievement of the recommended targets of >30 ng/mL after 12 months [63].

In CKD non-dialysis patients, this is not usually necessary, as according to our experience, 50,000 IU of cholecalciferol monthly is sufficient in most cases.

Even though these protocols demonstrated their efficiency, safety and simplicity, others can be used after validation with serum 25(OH)D, PTH, calcium, and phosphate levels.

Protocols using initially high loading doses and subsequent low dose displayed less efficiency and the risk for over dosing during the first weeks and under dosing thereafter has been reported [64,65].

It must be noted that the pharmaceutical vitamin D_3_ dosages that are available greatly differ from one country to another. In France, only drops (300 IU/drop) or calcium + vitamin D_3_ combinations are available for daily supplementation, while 80,000, 100,000 and 200,000 IU vials are available as spaced-out doses. This is the reason why monthly doses are most often prescribed in France. However, a recent meta-analysis of randomized controlled trials (RCTs) has shown that daily (or weekly) vitamin D_3_ doses are more efficient to reduce acute respiratory tract infections than monthly doses [66]. Several vitamin D experts have provided reasons why daily dosing is much better than bolus dosing [67,68], explaining why many of the trials that used bolus doses resulted in negative findings. Furthermore, while daily doses of 700–1000 IU/day reduce the risk of falls [69] and fractures [70] in the elderly, it was found in a RCT published in 2010 that elderly women who received a very large annual dose of 500,000 IU had more falls than those who received the placebo [71]. Recently, Bischoff-Ferrari et al. have shown in a RCT that frail elderly patients who received monthly doses of 60,000 IU (equivalent to 2000 IU/day) during one year sustained significantly more falls than other patients who were randomized to 24,000 IU per month (equivalent to 800 IU/day) [72]. As the same authors previously found no difference in terms of falls between women who received 800 IU/day or 2000 IU/day [73], their recent results suggest that higher monthly doses are more deleterious than beneficial for the risk of falls compared to moderate ones. Although such data do not exist in CKD patients treated or not treated by chronic dialysis, we take the above-mentioned results into account and we expect that daily vitamin D_3_ doses that are more suitable for clinical use than those currently available will be introduced in France in the near future.

## 5. Effect of Native Vitamin D Supplementation on CKD and Dialysis Patients

The main reported effects of vitamin D supplementation are displayed in Table 3. These effects depend on the vitamin D dosage, the type of vitamin D compounds, the duration of the study, and the studied population. One of the main expected effects is the lowering of serum PTH level. Hence, the results are not always positive. In a meta-analysis, Kandula et al. reported that nutritional vitamin D leads to increased 25(OH)D levels (mean + 24 ng/mL) without any hypercalcemia or hyperphosphatemia and with a decrease in serum PTH level (41% decrease), mostly in dialysis patients [74]. The mean dosage was 50,000 IU weekly during the first month; a lower dosage was used thereafter. Cupisti et al. and Alvarez et al. [75] reported mildly reduced serum PTH levels after vitamin D supplementation [76]. We have reported a decrease in SHPT in dialysis patients after systematic vitamin D supplementation during the predialysis period [77]. Novel modified-release calcifediol seems to have significant efficacy in decreasing PTH in CKD patients [78]. A recent randomized controlled trial (RCT) assessing short-term effects of ergocalciferol, weekly or monthly, during three months, failed to find a significant effect on PTH level [79]. However, comparing the different published regimens, Tangpricha et al. suggested that insufficient dosages (i.e., <100,000 U/month) may not be sufficient to achieve adequate replenishment, increased 1,25(OH)_2_D and decreased PTH [80]. We need to determine the dose range for responsiveness using ergocalciferol or cholecalciferol and different protocols (daily, weekly, or monthly administration). However, due to its shorter high life, ergocalciferol should not be prescribed on a monthly regimen. Massart et al. [56] and we [59] reported increased serum 1,25(OH)_2_D level after cholecalciferol supplementation. Seibeirt et al. confirmed these data and additionally did not find an increase in FGF-23 concentration after vitamin D supplementation [81].

Aytac et al. reported a favorable effect of high-dose cholecalciferol on cardiovascular and endothelial parameters of children with CKD [83] by using flow-mediated dilatation, arterial stiffness, homocysteine, and von Willebrand factor measurements. Karakas et al. confirmed that eight weeks of cholecalciferol improved the percentage of flow-mediated dilatation in dialysis with CKD patients [85]. In diabetic non-dialyzed patients using angiotensin-converting enzyme inhibitors, a decrease in proteinuria by adding native vitamin D was found by Kim et al. [82]. Meireless et al. reported in a RCT that cholecalciferol (50,000 twice weekly) promoted upregulation of CYP27B1 and VDR expression in monocytes and decreased serum IL-6 and C-reactive protein levels [84]. In a recent meta-analysis, Mann et al. reported a lack of significant effects of vitamin D supplementation on mortality [86].

Results of vitamin D trials vary for the general population and renal patients. The discrepancies may be due to differences in baseline serum 25(OH)D levels, vitamin D doses and treatment periods, adherence to supplementation, and VDR genetic polymorphisms [87].

## 6. Vitamin D Toxicity?

Opposite to vitamin D receptor activator (VDRA), nutritional vitamin D compounds are unlikely to induce hypercalcemia using a normal regimen because its 1α-hydroxylase activation is regulated by PTH, FGF-23 and 24-hydroxylase.Therefore, a serum 25(OH)D level up to 100 ng/mL is considered safe [1]. In the general population, daily vitamin D intakes >10,000 IU may be toxic because they lead to DBP saturation with an increase of free serum 25(OH)D [88]. In addition, toxicity has been observed for higher dosages (>40,000 U/day) [89]. Jacobus et al. reported eight cases of vitamin D intoxication that appear to have been caused by excessive vitamin D fortification of dairy milk with serum 25(OH)D >300 ng/mL [90].

Vitamin D toxicity is increased by higher calcium intake, calcitriol analogs, and adynamic bone disease in dialysis patients. The frequency of this toxicity is not known but appears very rare. Diagnosis mainly includes hypercalcemia with the risk for extraosseous calcification. Hypercalciuria is not frequently observed because calciuria is very low in CKD and dialysis patients. The native vitamin D compounds’ half-life is very long, approximatively two weeks, and toxicity should be treated for weeks. The physiopathology of hypercalcemia includes higher 1,25(OH)_2_D synthesis, higher intestinal absorption of calcium, and higher calcium release from bone. Close biological monitoring (serum PTH, calcium and phosphate levels), at least in dialysis patients, could prevent vitamin D toxicity.

## 7. Vitamin D_2_ or D_3_?

In 2008, Cavalier et al. reported some problems with 25(OH)D assays when measuring 25(OH)D_2_ [91]. These problems are now solved. Armas et al. reported that ergocalciferol displays a shorter half-life and is less potent compared with cholecalciferol with the same initial peak, but the serum 25(OH)D plateau decreased quickly after a few days using D_2_ and lasting 14 days using D_3_ [92]. Holick et al. showed that D_2_ and D_3_, given as daily doses, display the same efficiency in increasing serum 25(OH)D levels [93]. However, Oliveri et al., using the same loading and daily dose, report a superiority for D3 compounds [94].

More recently, Wetmore et al. reported that therapy with cholecalciferol, compared with ergocalciferol, is more effective at raising serum 25(OH)D in non-dialysis-dependent CKD patients using the same dosage (50,000 IU weekly) [95]. Lehmann et al. reported that vitamin D_3_ increases the total 25(OH)D concentration more than vitamin D_2_ and vitamin D_2_ supplementation was associated with a decrease in 25(OH)D_3_, which can explain the different effect on total 25(OH)D [96].

Ergocalciferol is mostly used in the United States. In other countries, such as France, cholecalciferol is the standard form, at least for CKD patients. For dialysis patients, we currently use 100,000 IU of oral cholecalciferol monthly.

## 8. VDRA for CKD and Dialysis Patients

As reported since the 1970s and mostly the 1980s, calcitriol is efficient for treating SHPT with a 50% decrease in baseline serum PTH values [16,97]. Even though cinacalcet is a commonly used therapy for resistant SHPT, calcitriol and analogs (which are VDRAs) remain the first-line therapy. VDRAs are very efficient in decreasing serum PTH level, but could lead to adynamic bone disease and risks for toxicity and hypercalcemia, as reported initially when the target serum PTH value was lower [16,97]. Toxicity risk is increased by higher dosages, concomitant prescriptions of oral calcium, high dialysate calcium concentrations (>1.5 mmol/L), and native vitamin D. Adynamic bone disease is suspected with low serum bone marker levels (bone-specific alkaline phosphatase). The consequences of hypercalcemia are well known and related to its cellular toxicity. This could justify close biological monitoring, especially when prescribing higher dosages. However, hypercalcemia and hyperphosphatemia are less frequent than in the 1980s because the serum PTH target has increased, from a normal value to two to nine times the upper limit of the assay, leading to reduce the VDRAs dosage. Prospective interpretation of serum PTH evolution is not easy, as when prescribing calcimimetics, and bone marker evolution could help with therapeutic adjustment.

It has been reported that oral bolus administration of VDRA three times weekly was more efficient than the intravenous route [98]. The intravenous route is much more expensive and should be used only when the oral route is not suitable, such as in the case of gastrointestinal malabsorption. Administration of calcitriol or analogs could be performed daily or using a bolus. It is most often administered three times weekly after dialysis. In France, the mean weekly dosage is <3 μg of alfacalcidol, which is equivalent to 1.5 μg of calcitriol.

VDRAs are clearly complementary with calcimimetics treatment to prevent hypocalcemia and improve PTH lowering [99]. The initial choice between these two therapies depends mainly on calcemia. When hypercalcemia occurs, tertiary HPT is suspected and calcimimetics could be the initial choice. In other cases, VDRAs could be the first choice for treating SHPT. In some large cohorts, mainly in the United States, it has been shown that dialysis patients treated with VDRAs displayed better outcomes [100,101,102,103]. A meta-analysis reported better survival for both CKD and dialysis patients treated with VDRAs [104].

A non-randomized prospective study performed in Japan reported lower mortality rates for dialysis patients treated with alfacalcidol [105]. We reported the same survival advantage in a French cohort of dialysis patients treated with alfacalcidol [45]. We do not know if this survival advantage is related to higher serum 1,25(OH)_2_D levels. At the moment, no interventional study showed any survival advantage for CKD and dialysis patients using native vitamin D supplementation. Tanaka et al. reported that infection-related mortality in Japan is reduced in patients receiving VDRA mostly intravenously [106]. In a recent meta-analysis of CKD, Li et al. reported that VDRAs reduced the incidence of cardiovascular events and reduced proteinuria, but resulted in an increased probability of hypercalcemia when paricalcitol was used [107]. In an observational study performed in Japan, it was reported that VDRA prescriptions during the late stages of CKD are associated with fewer cardiovascular diseases during the early dialysis stage [108]. Survival analysis in some observational cohorts from North America show advantages for patients treated with a synthetic analog (Paricalcitol), which is thought to be less hypercalcemic than calcitriol [100,102]. However, during treatment of SHPT, biological consequences, hypercalcemia and hyperphosphatemia were not different between paricalcitol and alfacalcidol [109]. Low-dose cholecalciferol in vitamin D-deficient HD patients and paricalcitol in cases of persistent SHPT have been reported to be efficient and to have no side effects [110].

Lou et al. reported a synergistic in vitro effect of 25(OH)D_3_ with 1α,25(OH_2_)D_3_ in Cyp27b1(−/−) cells that demonstrates the agonistic action of 25(OH)D3 of VDR [111].

In a recent study, in rat with SHPT, the correction of vitamin D deficiency effectively reversed the resistance to paricalcitol induction of CCAAT/Enhancer-binding-protein β (C/EBP-β) to suppress ADAM metallopeptidase domain 17 expression (also called tumor necrosis factor-α-converting enzyme) and parathyroid gland enlargement, reducing PTH by 50% [112].

Agarwal et al. recommend prescribing only VDRA because nutritional vitamin D did not treat SHPT or improve outcomes [113]. Zoccali et al. recently recommended that native compounds should not be prescribed to CKD patients treated with VDRA because there is no evidence indicating that native vitamin D has no biological effect beyond the calcitriol metabolite [114].

However, the correction of calcitriol deficiency to correct SHPT with VDRA does not correct nutritional vitamin D deficiency and all the health benefits of normal vitamin D status, unrelated to a calcitriol status, will not be provided.

## 9. Our Main Message

We think that native vitamin D supplementation should be the first line of therapy for SHPT prevention. The main reasons are summarized in Table 4, but once SHPT or tertiary hyperparathyroidism (HPT) is observed, adding VDRA and/or calcimimetics is justified. However, unanswered questions still exist (Table 5).

## 10. Conclusions

Vitamin D insufficiency, which involves both serum 25(OH)D and 1,25(OH)D levels, is generally observed in CKD and dialysis patients. The main consequence is SHPT, and vitamin D compounds remain the first-line therapy for its prevention and treatment.

Morbidity and mortality rates are associated with 25(OH)D insufficiency in CKD patients, but only VDRAs have been associated with better outcomes in large observational cohorts. Some questions remain unanswered about indication for serum vitamin D (25(OH)D and 1,25(OH)_2_D) measurements and about the real impact of these therapies on outcomes.

However, based on the recent published data, it appears justified to supplement CKD patients with 25(OH)D insufficiency and to use VDRAs for SHPT and hypocalcemia treatment. At last, large RCTs with clinically meaningful endpoints (fracture, hospitalization, parathyroidectomy, death) are still required to assess the usefulness of different vitamin D compounds for CKD and dialysis patients.

## Figures and Tables

**Table 1:** Causes and risk factors for 25(OH)D deficiency or insufficiency in CKD and dialysis patients.

- Age [22], female sex [18,23], adiposity [24] - Proteinuria [22] - Low physical activity [22] - Peritoneal dialysis [25] - Diabetes mellitus [23] - Reduced VDR [26] - Impaired 25(OH)D tubular reabsorption [27] - Reduced skin synthesis of vitamin D [28] - Calcineurin inhibitor prescriptions [18] - Reduction of the liver CYP450 isoform in SHPT [29]

**Table 2:** Association between vitamin D deficiency or insufficiency and outcomes for CKD and Dialysis populations.

- Secondary HPT [32] and high bone turnover markers [33] - Low bone mineral density [32,34] - Muscle weakness [35] and risk of falls [36] - Metabolic syndrome and obesity [37], insulin resistance [38] - Left ventricular hypertrophy and atherosclerosis [38] - Vascular calcification [39,40] and arterial stiffness [41] - Cognitive impairment [42] - Progression of kidney disease [43] - Mortality [43,44,45]

**Table 3:** Reported effects of vitamin D supplementation on CKD and dialysis patients.

- Serum PTH level decrease [74,75,76,78] - Serum 1,25(OH)_2_D level increase [56,59,81] - Reduced proteinuria [82] - Endothelial cardiovascular markers improvement [83] - Inflammation markers decrease [84]

**Table 4:** Why use supplements for vitamin D deficiency or insufficiency for CKD and dialysis patients?

- Low serum 25(OH)D level is reported in approximatively 90% of CKD and dialysis patients - 25(OH)D is the fuel for endocrine renal and cellular 1,25(OH)_2_D synthesis - 25(OH)D interacts with VDR in various target organs - Low vitamin D status leads to SHPT - 25(OH)D deficiency and insufficiency are associated with progression of renal disease, morbidity and mortality in CKD and dialysis patients - Nutritional compounds are inexpensive, not harmful, and are effective for preventing SHPT - Pleiotropic effects such as improvements in proteinuria and cardiovascular disease have been reported

**Table 5:** Unanswered questions.

- What is the optimal serum 25(OH)D target level according to different CKD stages and severity of SHPT? - Should we supplement patients with low serum PTH level? - What is the optimal 25(OH)D level for different outcomes (proteinuria, fractures, cancer, cardiovascular and immune disease . . .)? - Is it necessary to maintain native vitamin D supplements in patients treated with VDRAs? - Other than hypercalcemia, is there long-term toxicity associated with maintaining high serum 25(OH)D levels? - Are daily vitamin D doses better than weekly or monthly bolus doses?
